# Evaluating the impact of emergency department crowding on disposition patterns and outcomes of discharged patients

**DOI:** 10.1186/s12245-019-0223-1

**Published:** 2019-01-30

**Authors:** Mahshid Abir, Jason E. Goldstick, Rosalie Malsberger, Andrew Williams, Sebastian Bauhoff, Vikas I. Parekh, Steven Kronick, Jeffrey S. Desmond

**Affiliations:** 1 Department of Emergency Medicine, Acute Care Research Unit, Institute for Healthcare Policy and Innovation, University of Michigan, NCRC Bldg. 10 Rm G016, 2800 Plymouth Road, Ann Arbor, MI 48109-2800 USA; 2 RAND Corporation, Santa Monica, CA USA; 3 Mathematica Policy Research, Boston, MA USA; 4 Hackensack University Medical Center, Hackensack, NJ USA; 5 Department of Global Health and Population, Harvard T.H. Chan School of Public Health, Boston, MA USA; 6 Department of Internal Medicine, University of Michigan, Ann Arbor, MI USA; 7 Department of Emergency Medicine, University of Michigan, Ann Arbor, MI USA

**Keywords:** Emergency department crowding, Outcomes, Disposition decision-making

## Abstract

**Background:**

Crowding is a major challenge faced by EDs and is associated with poor outcomes.

**Objectives:**

Determine the effect of high ED occupancy on disposition decisions, return ED visits, and hospitalizations.

**Methods:**

We conducted a retrospective analysis of electronic health records of patients evaluated at an adult, urban, and academic ED over 20 months between the years 2012 and 2014. Using a logistic regression model predicting admission, we obtained estimates of the effect of high occupancy on admission disposition, adjusted for key covariates. We then stratified the analysis based on the presence or absence of high boarder patient counts.

**Results:**

Disposition decisions during a high occupancy hour decreased the odds of admission (OR = 0.93, 95% CI: [0.89, 0.98]). Among those who were not admitted, high occupancy was not associated with increased odds of return in the combined (OR = 0.94, 95% CI: [0.87, 1.02]), with-boarders (OR = 0.96, 95% CI: [0.86, 1.09]), and no-boarders samples (OR = 0.93, 95% CI: [0.83, 1.04]). Among those who were not admitted and who did return within 14 days, disposition during a high occupancy hour on the initial ED visit was not associated with a significant increased odds of hospitalization in the combined (OR = 1.04, 95% CI: [0.87, 1.24]), the with-boarders (OR = 1.12, 95% CI: [0.87, 1.44]), and the no-boarders samples (OR = 0.98, 95% CI: [0.77, 1.24]).

**Conclusion:**

ED crowding was associated with reduced likelihood of hospitalization without increased likelihood of 2-week return ED visit or hospitalization. Furthermore, high occupancy disposition hours with high boarder patient counts were associated with decreased likelihood of hospitalization.

## Background

Emergency departments (ED) are a critical component of the healthcare safety net, available 24 h a day, 7 days a week, to all who require care. Over the past 20 years, the number of ED visits in the United States (U.S.) has increased at roughly twice the rate of the population growth [1, 2], whereas the number of non-rural EDs has decreased by 27% [3]. As a result, EDs throughout the nation are crowded [4] and patients have less access to timely emergency care [5–7].

ED crowding is a major concern because it is associated with adverse clinical outcomes. Studies have demonstrated that crowding correlates with increased morbidity [8, 9], mortality [10–17], delays in treatment [13, 18–25], non-compliance with treatment guidelines [26–28], provider errors [29–31], length of stay [12, 32–34], cost [12, 35, 36], elopement, [5, 6, 37–40] return visits and readmissions [15, 41], and decreased patient satisfaction [42, 43].

It stands to reason that ED crowding may also affect physicians’ disposition decisions. The decision to admit or discharge a patient from the ED is influenced by patient-specific factors (diagnosis, medical condition, preference), physician-specific factors (knowledge, experience, self-defined risk thresholds), and hospital-specific factors (existing care protocols and medical resources) [44]. For patients with serious conditions, the decision is straightforward. In other cases, however, the decision is not so clear and the physician must carefully weigh the above factors and, if analytical evaluation does not produce an answer, make a decision based on clinical gestalt. Consciously or not, the stress of crowding may influence this gestalt [44].

The disposition decision to admit or discharge a patient is one of the most important decisions made by an emergency physician. Admitting a patient who does not need to be admitted exposes them to unnecessary medical testing, treatments, and expenses. However, an overly optimistic assessment of a patient’s condition and subsequent discharge can lead to negative clinical outcomes [45] and law suits [46]. Thus, it is imperative that we understand the factors that influence ED disposition decisions. Further, crowding may affect a patient’s willingness to wait in the waiting room during high occupancy periods or to stay if offered admission.

Only a handful of studies have investigated the effect of ED crowding on patient disposition decisions. A recent study of transient ischemic attack (TIA) and minor stroke patients found that crowding was associated with an increased likelihood of admission [47]. A study of pediatric ED patients with asthma and gastroenteritis found that crowding was associated with a lower likelihood of admission and lower frequency of return visits within 48 h [48]. To date, only one study has evaluated the effect of ED crowding on disposition of all-comers (i.e., regardless of diagnosis) [49]. This was a relatively small study at a community hospital ED and found no association between crowding and the likelihood of a patient’s admission versus discharge. The findings from this study need to be confirmed in other settings. Given these mixed findings from a limited number of pertinent studies, our study is important in helping to fill the knowledge gap regarding the effect of ED crowding on disposition patterns and subsequent outcomes for discharged patients.

In this study, we sought to determine the relationship between high ED occupancy—including in settings with and without boarders—and disposition decisions. Secondly, we sought to determine if patients discharged home during high ED occupancy hours were more likely to revisit the ED and require subsequent hospitalization than patients who were discharged during non-high occupancy ED hours. Based on the experience of the admitting hospitalist service at our institution, we hypothesized that, while controlling for other relevant case features, ED physicians will be less likely to admit a patient during high occupancy hours.

## Methods

### Study design and setting

We conducted a retrospective analysis of coded data from electronic health records (EHRs) of patients evaluated at an adult ED (where the majority of patients are aged 18 years and older) over a 20-month period between the years 2012 and 2014.

The study site is an 86-bed ED located in a tertiary care teaching hospital in an urban setting. The annual ED patient volume was approximately 85,000 during the study period. The ED is staffed around the clock with board-certified attending emergency physicians and supervised physician assistants and trainee resident physicians.

This study was approved by the Institutional Review Board at our institution.

### Patients

We used data from all patients who received care in the ED during the study period.

### Methods and measurements

The primary outcome variable was patient disposition; whether or not the patient was admitted (including either to observation or inpatient settings) or discharged. As secondary outcomes, we also examined, for those who were not admitted, the indicator of return visit to the ED within 2 weeks of discharge and, among those that did return within 2 weeks, we examined whether they were admitted during their return visit.

The primary exposure was whether or not the patient’s disposition was decided during a high occupancy hour. Because occupancy can vary substantially throughout a given day, the determination of high occupancy was based on the total occupancy (in terms of patient-per-bed) during the hour of disposition decision. To convert this occupancy to a proportion, we used the total number of available beds in our ED in each hour as the denominator. At our facility, the number of available beds fluctuates in a predictable manner during the course of the day (i.e., some beds are closed on a pre-planned basis at the same time each day), hence the occupancy denominator is variable during a 24-h cycle. We used the total number of available beds in our ED in each hour as the denominator. ED occupancy in each hour was compared to that same hour on all other days in the data set, and all hours in the top decile of that list were designated high occupancy hours, producing a binary indicator of high occupancy. We compared each hour to only the same hour across all days. For example, if an individual’s disposition decision occurred at 7:55 pm, then the ED occupancy between 7:00–8:00 pm on that day was compared to the occupancy during the same timeframe on all other days in the dataset to determine if it was in the top decile. In this way, each hour of a given day is marked as high occupancy independently of the other hours of the day. We took this approach, instead of comparing each hour to all other hours in a given day, because we noted that when we compared to all other hours, some AM hours were never marked as crowded, since they were always at a lower occupancy than other more typically crowded hours of the day. Further, comparing each hour to the same hour across all days in the dataset will allow the impact from factors such as time during a shift (e.g., beginning, middle, and end of a shift) and the time before and around sign-out to be held constant—as such factors may impact disposition decision-making independent of ED occupancy levels or presence or absence of boarders.

All patients who were placed in a bed in the ED are captured as part of the total occupancy (regardless of whether they left with or without being seen). However, only patients who had a discharge or admission diagnosis were included in the analysis of disposition decisions and assessment of the study outcomes.

ED occupancy fluctuates according to the time of day, day of the week, and the time of year. We chose to measure the ED occupancy every hour to capture this variability. The more granular the measurements of crowding, the more information is preserved to study the effects of crowding on the outcomes of interest. In particular, occupancy that is measured at a daily level may mask much of the variation in occupancy that occurs within a 24-h period [50]. For example, a patient whose disposition decision is made at 7:00 am when the ED is not crowded is not affected by crowding that occurs later in the day. However, a model that measures occupancy in 1-hour increments would allow assessing whether patients’ disposition decisions occurred during a crowded period. Furthermore, from a pragmatic standpoint, a 1-hour interval likely better reflects the reality of working in the ED. The physician is unlikely to be aware of minute-to-minute fluctuations in the ED occupancy, or 24-h fluctuations. As a final rationale, a supplemental analysis of data from our ED showed that approximately 55.2% of the total variation in occupancy was due to hour of day, 22.4% was due to daily variation, and 22.4% was unexplained variation (Appendix).

The other exposure of interest in this analysis was the presence of ED boarders. ED boarding is defined as holding an admitted patient in the ED until an inpatient bed becomes available [51]. To identify hours with high boarder patient counts, we first calculated boarding time for each encounter as the difference between ED disposition and inpatient (IP) floor arrival time. Because these two times are never exactly equal (i.e., every encounter has some non-zero boarding time), we only counted a patient as a boarder if his boarding time was above the average of 4.29 h for our sample. Because this still identified > 90% of all hours as having boarders, we further only classified an hour as having boarders if that hour had an above average number of boarders (8.69). Using this method, we increased the likelihood of identifying high occupancy ED hours that correspond with low IP capacity.

### Statistical analysis

We began with descriptive analyses of the sample based on patient demographics, severity of illness, payer source, day of week and arrival season, number of ED to ED transfer denials on arrival day, percentage with boarders at disposition decision hour, and percentage with disposition decided during a high occupancy hour. This analysis was then stratified by those who were (vs. were not) admitted, and two-sample comparisons were made to conduct an unadjusted evaluation of variables associated with admission.

Using a logistic regression model predicting admission, we obtained estimates of the effect of high occupancy on admission disposition, adjusted for age, race, gender, severity of illness (using facility level billing [52] and the Charlson index [53, 54]), payer source, day of week, season, presence of boarders, and number of ED-ED transfer denials. The Charlson-Deyo index is a commonly used co-morbidity index, assessed from each patient’s discharge diagnosis ICD-9 codes, that is predictive of hospital mortality [53, 54]. Facility billing level is a CMS outpatient payment coding system analogous to inpatient Diagnosis Related Groups (DRGs). It is used to reflect the volume and intensity of resources utilized by the facility to provide patient care [52]. A measure of high boarder patient counts was also included in all models. In a second analysis, we explored the role of high boarder patient counts as a possible modifier of the relationship between high occupancy and admission by presenting models stratified by presence of high boarder patient counts. We also fit logistic regression models to examine how high occupancy at disposition decision hour correlates with 14-day return ED visits for those who were discharged and, among those that did return, whether they were admitted during the return ED visit.

## Results

Our data set included 111,529 ED visits, and 12,735 (11.2%) disposition decisions were made during a high occupancy hour. Descriptive analysis of the study population at the encounter-level is shown in Table 1. The participants are predominantly Caucasian (75.4%) or African American (16.7%), between the ages of 25 and 64 (63.0%), and presented due to either emergent or urgent (87.1%) needs. Those who were ultimately admitted were more likely to be Caucasian, were older, tended to have higher acuity levels, were more likely to be on Medicare, and had high Charlson Index values (all *p* < .001). Presence of boarders at disposition decision hour was more likely among those who were admitted (*p* < .001). Mean number of ED-ED transfer denials on arrival day was not significantly different between those who were versus were not admitted (*p* = .058). Statistically significant differences were found with regard to arrival day of the week and arrival season. See Tables 2 and 3 for descriptive analyses of the study population stratified to whether or not there were boarders in the ED.

**Table 1:** Patient demographics “encounter-level”

	Overall	Admission	No admission	*p* value
Female (*n*, %)	50,609 (45.4%)	19,895 (48.6%)	30,714 (43.5%)	< .0001
Race (*n*, %)				
White/Caucasian	84,095 (75.4%)	32,663 (79.8%)	51,432 (72.9%)	< .0001
Black/African American	18,674 (16.7%)	5981 (14.6%)	12,693 (18.0%)	
Asian	3490 (3.1%)	842 (2.1%)	2648 (3.8%)	
Other/unknown	5268 (4.7%)	1456 (3.6%)	3812 (5.4%)	
Age category (years) (*n*, %)				
0–24	17,772 (15.9%)	2611 (6.4%)	15,161 (21.5%)	< .0001
25–44	34,428 (30.9%)	8454 (20.6%)	25,974 (36.8%)	
45–64	35,825 (32.1%)	15,440 (37.7%)	20,385 (28.9%)	
65–84	11,993 (10.8%)	7064 (17.3%)	4929 (7.0%)	
85+	11,509 (10.3%)	7373 (18.0%)	4136 (5.9%)	
Acuity level (*n*, %)				
Resuscitation	1290 (1.2%)	1210 (3.0%)	80 (0.1%)	< .0001
Emergent	45,619 (40.9%)	25,167 (61.5%)	20,452 (29.0%)	
Urgent	51,489 (46.2%)	14,178 (34.6%)	37,311 (52.9%)	
Non-urgent	11,960 (10.7%)	378 (0.9%)	11,582 (16.4%)	
Minor	1169 (1.0%)	9 (0.0%)	1160 (1.6%)	
Primary payer (*n*, %)				
BCBS	44,191 (39.6%)	13,563 (33.1%)	30,628 (43.4%)	< .0001
Commercial	25,308 (22.7%)	7288 (17.8%)	18,020 (25.5%)	
Medicaid	4805 (4.3%)	2120 (5.2%)	2685 (3.8%)	
Medicare	27,754 (24.9%)	16,343 (39.9%)	11,411 (16.2%)	
Military	536 (0.5%)	201 (0.5%)	335 (0.5%)	
Self-Pay	7816 (7.0%)	1238 (3.0%)	6578 (9.3%)	
Workers comp	1117 (1.0%)	189 (0.5%)	928 (1.3%)	
Arrival day (*n*, %)				
Sunday	14,717 (13.2%)	4926 (12.0%)	9791 (13.9%)	< .0001
Monday	17,495 (15.7%)	6729 (16.4%)	10,766 (15.3%)	
Tuesday	16,106 (14.4%)	6008 (14.7%)	10,098 (14.3%)	
Wednesday	15,974 (14.3%)	6128 (15.0%)	9846 (13.9%)	
Thursday	15,725 (14.1%)	5905 (14.4%)	9820 (13.9%)	
Friday	16,577 (14.9%)	6269 (15.3%)	10,308 (14.6%)	
Saturday	14,933 (13.4%)	4977 (12.2%)	9956 (14.1%)	
Arrival season (*n*, %)				
Spring	18,696 (16.8%)	7046 (17.2%)	11,650 (16.5%)	0.004
Summer	35,128 (31.5%)	12,684 (31.0%)	22,444 (31.8%)	
Autumn	33,537 (30.1%)	12,351 (30.2%)	21,186 (30.0%)	
Winter	24,166 (21.7%)	8861 (21.6%)	15,305 (21.7%)	
Charlson index (mean, std)	0.6 (0.9)	0.9 (1.1)	0.4 (0.7)	< .0001
Number of denials arrival day (mean, std)	1.5 (1.9)	1.5 (1.9)	1.4 (1.9)	0.058
Boarders at disposition hour (*n*, %)	50,943 (45.7%)	19,907 (48.6%)	31,036 (44.0%)	< .0001

**Table 2:** Demographics (encounter-level)—stratified to with boarders only

	Overall	Admission	No admission	*p* value
Female (*n*, %)	22,575 (44.3%)	9519 (47.8%)	13,056 (42.1%)	< .0001
Race (*n*, %)				
White/Caucasian	38,552 (75.7%)	15,903 (79.9%)	22,649 (73.0%)	< .0001
Black/African American	8559 (16.8%)	2911 (14.6%)	5648 (18.2%)	
Asian	1479 (2.9%)	393 (2.0%)	1086 (3.5%)	
Other/unknown	2353 (4.6%)	700 (3.5%)	1653 (5.3%)	
Age category (years) (*n*, %)				
0–24	7338 (14.4%)	1192 (6.0%)	6146 (19.8%)	< .0001
25–44	15,508 (30.4%)	3950 (19.8%)	11,558 (37.2%)	
45–64	16,670 (32.7%)	7552 (37.9%)	9118 (29.4%)	
65–84	5796 (11.4%)	3543 (17.8%)	2253 (7.3%)	
85+	5631 (11.1%)	3670 (18.4%)	1961 (6.3%)	
Acuity level (*n*, %)				
Resuscitation	567 (1.1%)	534 (2.7%)	33 (0.1%)	< .0001
Emergent	21,673 (42.5%)	12,388 (62.2%)	9285 (29.9%)	
Urgent	23,453 (46.0%)	6819 (34.3%)	16,634 (53.6%)	
Non-urgent	4810 (9.4%)	163 (0.8%)	4647 (15.0%)	
Minor	440 (0.9%)	3 (0.0%)	437 (1.4%)	
Primary payer (*n*, %)				
BCBS	19,897 (39.1%)	6464 (32.5%)	13,433 (43.3%)	< .0001
Commercial	11,260 (22.1%)	3509 (17.6%)	7751 (25.0%)	
Medicaid	2237 (4.4%)	1034 (5.2%)	1203 (3.9%)	
Medicare	13,422 (26.3%)	8146 (40.9%)	5276 (17.0%)	
Military	250 (0.5%)	100 (0.5%)	150 (0.5%)	
Self-Pay	3345 (6.6%)	552 (2.8%)	2793 (9.0%)	
Workers comp	532 (1.0%)	102 (0.5%)	430 (1.4%)	
Arrival day (*n*, %)				
Sunday	1009 (2.0%)	364 (1.8%)	645 (2.1%)	< .0001
Monday	7575 (14.9%)	3139 (15.8%)	4436 (14.3%)	
Tuesday	9714 (19.1%)	3764 (18.9%)	5950 (19.2%)	
Wednesday	10,080 (19.8%)	3963 (19.9%)	6117 (19.7%)	
Thursday	9924 (19.5%)	3824 (19.2%)	6100 (19.7%)	
Friday	9563 (18.8%)	3756 (18.9%)	5807 (18.7%)	
Saturday	3078 (6.0%)	1097 (5.5%)	1981 (6.4%)	
Arrival season (*n*, %)				
Spring	9820 (19.3%)	3981 (20.0%)	5839 (18.8%)	0.006
Summer	12,796 (25.1%)	4897 (24.6%)	7899 (25.5%)	
Autumn	15,938 (31.3%)	6208 (31.2%)	9730 (31.4%)	
Winter	12,389 (24.3%)	4821 (24.2%)	7568 (24.4%)	
Charlson index (mean, std)	0.6 (0.9)	0.9 (1.1)	0.4 (0.7)	< .0001
Number of denials arrival day (mean, std)	2.1 (2.1)	2.1 (2.1)	2.1 (2.1)	< .0001

**Table 3:** Demographics (encounter-level)—stratified to without boarders only

	Overall	Admission	No admission	*p* value
Female (*n*, %)	28,034 (46.3%)	10,376 (49.3%)	17,658 (44.6%)	< .0001
Race (*n*, %)				
White/Caucasian	45,543 (75.2%)	16,760 (79.7%)	28,783 (72.8%)	< .0001
Black/African American	10,115 (16.7%)	3070 (14.6%)	7045 (17.8%)	
Asian	2011 (3.3%)	449 (2.1%)	1562 (3.9%)	
Other/unknown	2915 (4.8%)	756 (3.6%)	2159 (5.5%)	
Age category (years) (*n*, %)				
0–24	10,434 (17.2%)	1419 (6.7%)	9015 (22.8%)	< .0001
25–44	18,920 (31.2%)	4504 (21.4%)	14,416 (36.5%)	
45–64	19,155 (31.6%)	7888 (37.5%)	11,267 (28.5%)	
65–84	6197 (10.2%)	3521 (16.7%)	2676 (6.8%)	
85+	5878 (9.7%)	3703 (17.6%)	2175 (5.5%)	
Acuity level (*n*, %)				
Resuscitation	723 (1.2%)	676 (3.2%)	47 (0.1%)	< .0001
Emergent	23,946 (39.5%)	12,779 (60.8%)	11,167 (28.2%)	
Urgent	28,036 (46.3%)	7359 (35.0%)	20,677 (52.3%)	
Non-urgent	7150 (11.8%)	215 (1.0%)	6935 (17.5%)	
Minor	729 (1.2%)	6 (0.0%)	723 (1.8%)	
Primary payer (*n*, %)				
BCBS	24,294 (40.1%)	7099 (33.7%)	17,195 (43.5%)	< .0001
Commercial	14,048 (23.2%)	3779 (18.0%)	10,269 (26.0%)	
Medicaid	2568 (4.2%)	1086 (5.2%)	1482 (3.7%)	
Medicare	14,332 (23.7%)	8197 (39.0%)	6135 (15.5%)	
Military	286 (0.5%)	101 (0.5%)	185 (0.5%)	
Self-Pay	4471 (7.4%)	686 (3.3%)	3785 (9.6%)	
Workers comp	585 (1.0%)	87 (0.4%)	498 (1.3%)	
Arrival day (*n*, %)				
Sunday	13,708 (22.6%)	4562 (21.7%)	9146 (23.1%)	< .0001
Monday	9920 (16.4%)	3590 (17.1%)	6330 (16.0%)	
Tuesday	6392 (10.6%)	2244 (10.7%)	4148 (10.5%)	
Wednesday	5894 (9.7%)	2165 (10.3%)	3729 (9.4%)	
Thursday	5801 (9.6%)	2081 (9.9%)	3720 (9.4%)	
Friday	7014 (11.6%)	2513 (11.9%)	4501 (11.4%)	
Saturday	11,855 (19.6%)	3880 (18.4%)	7975 (20.2%)	
Arrival season (*n*, %)				
Spring	8876 (14.7%)	3065 (14.6%)	5811 (14.7%)	0.677
Summer	22,332 (36.9%)	7787 (37.0%)	14,545 (36.8%)	
Autumn	17,599 (29.0%)	6143 (29.2%)	11,456 (29.0%)	
Winter	11,777 (19.4%)	4040 (19.2%)	7737 (19.6%)	
Charlson index (mean, std)	0.5 (0.9)	0.9 (1.0)	0.3 (0.7)	< .0001
Number of denials arrival day (mean, std)	0.9 (1.5)	0.9 (1.6)	0.9 (1.5)	0.183

Logistic regression showed that, in the overall sample (not stratified by presence/absence of boarders), disposition decision during a high occupancy hour decreased the odds of admission (OR = 0.93, 95% CI: [0.89, 0.98]), and the presence of high boarder patient counts increased the odds of admission (OR = 1.10, 95% CI: [1.06, 1.13]). In the stratified model including only disposition decisions occurring in the presence of high patient boarder counts, disposition during a high occupancy hour decreased the odds of admission (OR = 0.90, 95% CI: [0.84, 0.96]). Restricting only to arrivals occurring during periods with low boarder patient counts, high occupancy was not associated with odds of admission (OR = 0.96, 95% CI: [0.90, 1.02]).

**Fig. 1:**
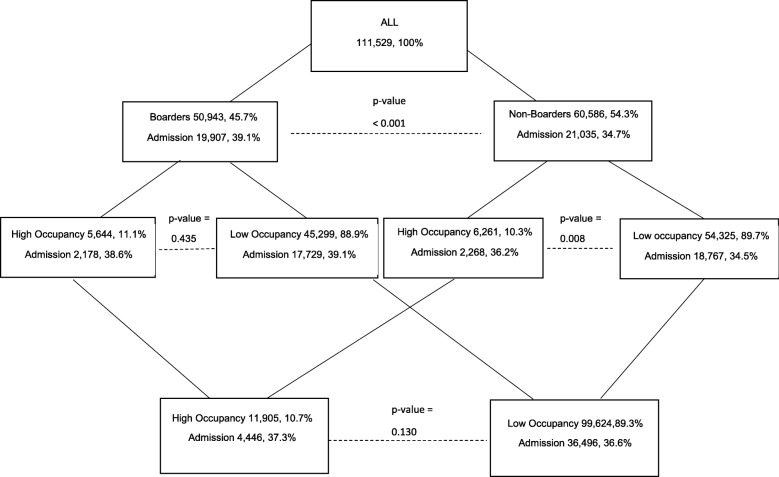
Stratification of “admitted during initial ED visit”

With regard to 14-day return ED visit, among those who were not admitted, high occupancy was not associated with increased odds of return in the combined sample (OR = 0.94, 95% CI: [0.87, 1.02]), in the with-boarders sample (OR = 0.96, 95% CI: [0.86, 1.09]), or in the no-boarders sample (OR = 0.93, 95% CI: [0.83, 1.04]). Among those who were not admitted and who did return within 14 days, disposition during a high occupancy hour on the initial ED visit was not associated with a statistically significant increased odds of hospitalization in the combined sample (OR = 1.04, 95% CI: [0.87, 1.24]), in the with-boarders sample (OR = 1.12, 95% CI: [0.87, 1.44]), or in the no-boarders sample (OR = 0.98, 95% CI: [0.77, 1.24]) (Fig. 1).

## Discussion

To the best of our knowledge, this is one of only two studies to examine the association between crowding and the disposition of all patients presenting to an ED [49]. We hypothesized that crowding would raise the threshold for admission based on the anecdotal experience of our institution’s hospitalist service. We found that, overall, patients whose disposition decisions were made during a high occupancy ED hour were less likely to be admitted to the hospital. This remained true after controlling for key covariates, including patient acuity and disease severity, daily denials of ED to ED transfers, day of the week, and season. Presence of high boarder patient counts during disposition decision hour was associated with increased odds of hospitalization. After stratifying by the presence or absence of high patient boarder counts, in order to evaluate periods that are more likely to correspond with high vs. low IP occupancy, respectively, we found that in the presence of high ED boarder counts, patients whose disposition decisions were made during high occupancy were less likely to be admitted. In the absence of high ED boarder patient counts, high occupancy did not affect the odds of admission. The fact that high boarder counts in high occupancy periods was associated with decreased odds of hospitalization, while high boarder counts without high occupancy was associated with increased odds of hospitalization, may indicate that it is the “in process” patients in a crowded ED that impact disposition decisions.

In the ED under study, nurse staffing increases with increased ED volume, and a physician’s assistant or resident is added during times where high volume is anticipated; however, attending physician staffing does not change based on volume.

The notion that crowding decreases the probability of admission raises an important question: When physicians are under pressure to see many patients in a crowded ED, do they provide suboptimal care and discharge patients who ought to be admitted? We investigated this by analyzing return visits to the ED within 2 weeks of discharge during a high occupancy hour and subsequent admissions during the return ED visit. We found that there was no significant relationship between high occupancy and 2-week return ED visits. This remained true regardless of the presence or absence of boarders. Among those who did return, disposition during a high occupancy hour during the initial visit was not associated with hospital admission in the combined sample, in the with-boarders sample, or in the no-boarders sample.

The literature evaluating whether patients seen during crowded conditions return to the ED and subsequently get hospitalized more often than patients seen during less crowded times shows mixed results. One study showed that crowding was associated with increase in hospital admission during return ED visits [14]. Looking at a composite outcome of 72-h returns, radiology over-readings, and quality improvement cases, Bernstein et al. found that patients with these endpoints were more likely to have initially been examined in the ED during periods of crowding [55]. A more recent study by the same author, at a different institution, did not find an association between crowding on the first ED visit and probability of admission during a return visit within 72 h [56].

For the primary outcome under study (i.e., the impact of ED crowding on disposition decisions), we believe that the 2-week window for assessing return ED visits and hospitalizations (as opposed to a 30-day return visit window) is more clinically meaningful. Return ED visits and hospitalizations during the 3rd and 4th week post-ED discharge may be due to other underlying reasons, or reasons unrelated to the sentinel ED visit. A 72-h window, on the other hand, may lead to missing some return ED visits related to the disposition decision during the sentinel ED visit.

There is currently no single, consensus measure of ED crowding [57]. In a systematic review, Hwang et al. identified 71 different measures in the medical literature, many of which had moderate to good correlation with validation criteria. Early studies included surveys of ED providers and simple measures of census and ED boarding. Later articles focused on the development of multidimensional measures that incorporate real-time census, staffing, patient acuity, and hospital variables. Multidimensional measures have been criticized for their lack of scalability at institutions other than the one where they were developed [58]. Hwang et al. concluded that simple measures, consisting of patient counts and time intervals, which affect clinical outcomes, are superior to complex, multidimensional measures [57].

In keeping with these findings, we chose to use ED occupancy rate to measure crowding because it is simple, valid, and associated with numerous clinical outcomes. ED occupancy rate is the ratio of the total number of patients in the ED to the total number of ED treatment rooms per hour. Several validation studies have demonstrated that ED occupancy rate is equal to, if not better than, other measures at predicting key surrogate indicators of crowding like clinician opinion of crowding, [59–61] patients who left without being seen, and ambulance diversions [62]. Furthermore, studies have shown that ED occupancy affects clinical outcomes such as morbidity [16] and mortality [15, 17], delay in treatment [21, 29, 34], compliance with treatment guidelines [27], provider errors [30, 32, 33], and patient satisfaction [43, 44]. Finally, two separate panels of experts rated ED occupancy highly as a measure of crowding, which suggests it has face validity [60, 63, 64].

Our findings indicate that at one academic, urban ED crowding is associated with reduced likelihood of hospital admission and that those that are discharged during such periods are not more likely to return to the ED within 2 weeks of discharge. Further, those that do return are not more likely to be admitted when compared to those that are discharged during non-crowded periods. High occupancy disposition hours with high boarder patient counts were associated with decreased likelihood of hospital admission. We observed that high boarder counts in times of non-high occupancy was associated with increased likelihood of hospitalization, indicating that perhaps it is the patients actively undergoing evaluation in the ED that may be impacting disposition decisions as opposed to those who are boarding after completed evaluation. This may also indicate that the high hospital occupancy—leading to greater number of boarders in the ED—may not significantly impact disposition decisions.

These results may indicate that practices during crowding in this ED, including a higher threshold for hospitalization, may lend themselves to safe patient disposition and spare unnecessary hospitalizations. These results point to a need to further evaluate health system level factors that influence physician disposition practices during high occupancy periods. Future research needs to be conducted to evaluate this study’s outcomes in other EDs across the USA in order to assess whether similar associations emerge. Furthermore, studies using both quantitative and qualitative data in this and other EDs will need to assess how disposition practices change during crowded periods and how such changes may impact the observed outcomes. Such work could inform the development of admission decision tools that can improve ED disposition practices even during non-crowded times.

## Limitations

The study limitations include general limitations pertaining to the use of coded administrative data, including the quality of coding, data validity, and generalizability of results. This study was performed at a single adult, academic, and urban ED that may affect the generalizability of its findings. Similar studies will need to be conducted using data from other EDs in order to assess the validity of our findings in alternate settings.

There are limitations associated with 14-day return ED visits post-discharge that should be noted. First, a 14-day return ED visit could be unrelated to the sentinel ED visit given the 2-week time elapsed. Second, return ED visits to other hospitals would not be captured and may bias the findings. Our dataset does not include information on ED visits and hospitalizations to other facilities in the state. If patients went to other EDs due to our ED being crowded or went to other facilities after discharge from our ED, we would not be able to account for those ED visits and hospitalizations.

Furthermore, the admission/discharge time stamp may not accurately reflect when a disposition decision was actually made by the ED physician. During busy periods in the ED, there may be a delay in when providers put in the admission/discharge order relative to when the decision is made. Also, although the study findings indicate that ED physicians may consider having a lower threshold for discharging patients during low occupancy periods, since we are not able to capture patients who may have sought care at other hospitals, these results should be used with caution.

## Conclusion

Crowding is a major challenge faced by EDs across the USA. ED crowding is associated with adverse outcomes, but few studies have evaluated the effect of crowding on patient disposition decisions. Our findings indicated that at one academic, urban ED crowding was associated with reduced likelihood of hospital admission and that those who were discharged during such periods were not more likely to return to the ED within 2 weeks of discharge. Further, those that did return were not more likely to be admitted, when compared to those who were discharged during non-crowded periods. High occupancy disposition hours with high boarder patient counts were associated with decreased likelihood of hospital admission.

These results may indicate that practices during crowding in this ED, including a higher threshold for hospitalization, may lend themselves to safe patient disposition and spare unnecessary hospitalizations. The statistically significant study findings may be a function of the large sample size.

This question should be evaluated in other EDs to assess whether similar associations exist in other settings. Future studies should assess changes in practice patterns by ED physicians during periods of crowding. Specifically, qualitative research can help elucidate practice-based, decision-making, and operational factors underlying the observed findings in this study.

## Appendix

We used a linear random effects model to decompose the variance in hourly occupancy rates into variance due to hour of the day, variation due to day of year, and all other forms of variation (i.e., error variance). From this analysis, we found that approximately 55.2% of the total variation was due to hour of the day, 22.4% was due to daily variation, and 22.4% was unexplained variation.

**Table 4:** Decomposition of hourly occupancy variation based on the linear random effects model

Daily	75.00 (22.4%)
Hourly	184.79 (55.2%)
Unexplained	74.77 (22.4%)
Total	334.56 (100.0%)

## Abbreviations

CMS: Centers for Medicare and Medicaid Services
ED: Emergency department
EHRs: Electronic health records
IP: Inpatient
TIA: Transient ischemic attack
